# Zinc Oxide Nanoparticles (ZnO-NPs) Suppress Fertility by Activating Autophagy, Apoptosis, and Oxidative Stress in the Developing Oocytes of Female Zebrafish

**DOI:** 10.3390/antiox11081567

**Published:** 2022-08-13

**Authors:** Suzan Attia Mawed, Carlotta Marini, Mahmoud Alagawany, Mayada R. Farag, Rasha M. Reda, Mohamed T. El-Saadony, Walaa M. Elhady, Gian E. Magi, Alessandro Di Cerbo, Wafaa G. El-Nagar

**Affiliations:** 1 Zoology Department, Faculty of Science, Zagazig University, Zagazig 44519, Egypt; 2 School of Biosciences and Veterinary Medicine, University of Camerino, 62024 Matelica, Italy; 3 Poultry Department, Agriculture Faculty, Zagazig University, Zagazig 44519, Egypt; 4 Forensic Medicine and Toxicology Department, Faculty of Veterinary Medicine, Zagazig University, Zagazig 44519, Egypt; 5 Department of Fish Diseases and Management, Faculty of Veterinary Medicine, Zagazig University, Zagazig 44511, Egypt; 6 Department of Agricultural Microbiology, Faculty of Agriculture, Zagazig University, Zagazig 44511, Egypt

**Keywords:** zinc oxide nanoparticles, autophagy, apoptosis, oxidative stress, ovary, zebrafish

## Abstract

In vertebrates, the core mechanisms that control gametogenesis are largely multiple, complex, successive, and orchestrated by intrinsic and extrinsic factors. However, age, health status, and hormonal activity are important factors for good fertility; other intangible intracellular molecular mechanisms that manage oocyte development are still unclear. The present study was designed to elucidate the ultrastructure changes in the ovary in response to its exposure to zinc oxide nanoparticles (ZnO-NPs) and to explore the role of autophagy and apoptosis during egg maturation and ovulation on the fertility of female zebrafish. In our study, ZnO-NPs could induce cytotoxicity in the maturing oocyte by activating autophagy and apoptosis in a caspase-dependent manner and could induce oxidative stress by generating reactive oxygen species (ROS) that elevated the mutated ovarian tP53 protein. Simultaneously, necroptosis developed, mimicking the features of apoptosis and necrosis. Collectively, ZnO-NPs created a suitable necrotic environment that led to follicular developmental retardation that altered oocyte ovulation and reduced fecundity of female zebrafish.

## 1. Introduction

Recently, nanoparticles (NPs) have attracted a lot of attention in the research community because they have greater chemical, physical, and biological properties than their bulky counterparts [1,2,3]. Zinc oxide nanoparticles (ZnO-NPs), as one of the ionic metal oxide nanoparticles, have attracted interest because of their diverse physical and chemical properties, as well as their antibacterial properties against a wide spectrum of pathogenic bacteria [4]. Therefore, ZnO-NPs have been widely used in the industrial sector in optoelectronics, catalysts, cosmetics, pigments, and ceramics due to their unique characteristics and their ability to create various nanostructures [4,5]. ZnO-NPs are also used in biological and biomedical sectors because of their anti-inflammatory, wound-healing, antiseptic, and anti-cancer characteristics [6,7].

However, increasing the use of ZnO-NPs has unavoidably resulted in higher human and environmental exposures, as well as toxicological impacts on environmental species. Nano-ZnO particles, on the other hand, are easily bioaccumulated by aquatic creatures, where they cause harmful consequences [8,9]. Of these harmful consequences, ZnO-NPs have toxic effects on the female reproductive system (oocytes), as well as the developing embryos [10,11].

Normal female reproduction and fetal development are required for the survival of all species, including humans and animals. In the female ovary, two essential processes are independently induced and cooperate before fertilization: oocyte maturation and ovulation.

Successful maturation requires the combination of intrinsic and extrinsic factors and passes through different developmental stages that were described earlier in the zebrafish [12]. The first stage of oocyte development is named the primary oocyte or previtellogenic oocyte, followed by the vitellogenic oocyte where the oocytes become more enlarged and reached their maximum size, followed by the third step named the post-ovulatory follicle, which occurs after ovulation [13,14]. In the vertebrate ovary, granulosa cells (GCs) and theca cells formed the follicular cell (FCs) mass around the developing oocyte and they can provide it with essential amino acids, cholesterol, and other nutrients before the ovulation [15,16]. 

In the developing oocyte and follicular cells cytoplasm, macrophagy (here, named autophagy) is one of the most-programmed regular intrinsic factors that maintain cellular homeostasis and energy production during germ cell starvation and manages the oogenesis process [17,18]. Autophagy is a bulk subcellular degradation process that helps in recycling the unused cytosolic components for cell survival, differentiation, growth, and development [19,20].

For further clarification, increasing evidence declared that autophagy played an essential role in the proliferation and survival of granulosa cells [21,22,23], suggesting its crucial role during the oogenesis to embryogenesis transition of both invertebrates and vertebrates [18,24,25,26].

Recent works have investigated different mechanisms for the relationship between ZnO-NP toxicity and female reproductive performance. For example, some authors reported that ZnO-NPs have cytotoxic effects in mouse ovarian germ cells (OGCs); even at low concentrations, ZnO-NPs impeded meiosis and developing abilities in OGCs [11]. 

Furthermore, ZnO-NPs caused oxidative stress, developmental toxicity, and DNA damage to zebrafish embryos, resulting in significant embryo hatching delays and increased deformity after a 96-h exposure [8].

The adult female zebrafish has asynchronous ovaries, containing ova in different developmental stages, making it a suitable model for studying all the concepts related to ovarian development [27,28,29]. In our study, we used the female zebrafish as a model to study the effects of ZnO-NPs on cytotoxicity, apoptosis, and autophagy in the developing oocytes in vivo. 

To achieve this objective, we investigated the histological findings of the ovarian oocytes under a light and transmission electron microscope as well as the changes in gene expression in nano-ZnO-treated zebrafish ovarian tissue by Reverse transcription-quantitative polymerase chain reaction (RT-qPCR) and Western blot analysis.

## 2. Materials and Methods

### 2.1. ZnO-NPs Preparation and Characterization 

The chemical Zn-NPs (Che-Zn-NPs) were prepared as follows; 12 g of Zn (NO_3_)_2_·6H_2_O was dissolved in 1 L of distilled water then was stirred with NaOH (10%) solution on a hot plate magnetic stirrer for 25 min, then for 2 h at 70 °C. The mixed solution was cooled and filtrated [30]. 

The synthesized ZnO-NPs were characterized using several techniques. To determine the optical absorption spectra of ZnO-NPs, ultraviolet-visible spectroscopy (UV-vis) (LaxcoTM dual-beam spectrophotometer) was used. In addition, transmission electron microscope analysis (TEM, JEOL 1010, Shimadzu, Japan) was used to determine the morphological characteristics of ZnO-NPs, particularly their size and form. Dynamic light scattering analysis was used to measure the particle size in the colloidal solution of produced ZnO-NPs (DLS, Malvern Hills, UK). Zeta potential analysis was conducted to determine the surface charge of ZnO-NPs and their relative stability. The LC_50_ of ZnO-NPs was estimated in our laboratory and found to be 3.48 mg/L.

### 2.2. Animal Husbandry and Experiment Design

Adult female zebrafish (*Danio rerio*) (*n* = 180) were bought from a local fish supplier (Cairo, Egypt) and acclimatized for two weeks before the experiment. During the acclimatization and throughout the experiment, the females were kept in aerated water at 27.5 ± 1 °C in glass aquaria (80 × 40 × 30 cm, water capacity 60 L), 14 h light: 10 h dark, pH 6.7 ± 0.2 and dissolved oxygen 6.3 ± 0.5 mg/L. The fish were fed twice a day on *Artemia nauplii* (hatched shrimp’s eggs) [31]. Acclimatized females were randomly divided into three experimental groups (*n* = 60/group), each in triplicate (20 fish) in separate tanks. The first group was used as the control group (receiving no treatment), the second group, termed the T1 group, was exposed to 1/5^th^ of the estimated LC_50_ of ZnO-NPs (0.69 mg/L) in water daily for two weeks (15 days), while the third group termed the T2 group was exposed to 1/5th of the estimated LC_50_ of ZnO-NPs daily for one month (30 days). The experimental procedures were carried out at the Zoology Department, Faculty of Science, Zagazig University, Zagazig, Egypt.

### 2.3. Fertility, Fecundity, and Gonad Somatic Index Calculations

Fertility refers to females that can lay ovulated eggs when crossed with adult males, whereas fecundity measures the reproductive potential through the number of fertilized spawned eggs after mating [32]. Herein, tracing fertility and fecundity assessment was conducted with 10 fish from each group, one-to-one paired with untreated males every 5 days. The spawning ratio for fertility was calculated [33]. In addition, to detect fecundity after mating between the females and males, spawned eggs were collected and counted after one hour of light in the morning. Data on fertility and fecundity was obtained from triplicate readings. The gonad somatic index (GSI) was calculated every five days. GSI is an indicator of reproductive capacity, commonly used to quantify the reproductive condition in fish (GSI = Gonad weight/Bodyweight ×100).

### 2.4. Determination of Zinc Residues in the Whole Fish Body

The bodies of female zebrafish (6 females/sample) were digested using acids [34]. In a clean screw-capped glass bottle, one gram of each sample was digested with 4 mL of digestion solution (perchloric/nitric acid, 1:1). Initial digestion was carried out at room temperature for 24 h, followed by heating for 2 h at 110 °C.

After cooling, deionized water was added, and the solution was warmed to expel nitrous gases for one hour in a water bath. Then, the digests were filtered using Whatman No. 1 filter and diluted to 25 mL with deionized water [35]. The resultant solution was then analyzed by a flame atomic absorption spectrophotometer (FAAS). 

### 2.5. Histological Assessments and Transmission Electron Microscope (TEM) Preparations

For histological observations, the females were anesthetized in ice water and fixed as a whole-mount for 24 h at room temperature in Bouin’s solution to preserve the ovary oocytes before dissection. Dissected fixed tissues were dehydrated and embedded in paraffin wax and sectioned transversely at 5 µm thicknesses (Leica Model, RM2125 RTS, Biosystems, Deer Park, USA). Sections were stained with hematoxylin and eosin [36]. The average number of oocytes at different stages was calculated to detect the percentage for each type per slide in triplicate. 

For TEM observations, dissected ovaries from the three experimental groups were fixed in a mixture of 2.5% glutaraldehyde and 2.5% paraformaldehyde in 0.1 M Na-cacodylate buffer (pH = 7.3) for 24 h at 4 °C. After rinsing in 0.1 M Na-cacodylate buffer, the fixed samples were post-fixed at room temperature in 1% osmium tetroxide. After that, graded series of ethanol were used for the dehydration of samples. Finally, the tissues were embedded in an Epon-Araldite mixture. Semi-thin sections (1.5 mm thickness) were cut using a Reichert ultra-microtome. Ultrathin sections were stained with toluidine blue contrasted in 50% alcohol-uranyl acetate solution and lead citrate, then examined with a Philips EM 400 electron microscope (Philips, INCAEDX, Oxford, UK) at the Faculty of Science, Alexandria, Egypt. 

### 2.6. RNA Isolation and Quantitative Real-Time PCR (qRT-PCR)

Total RNA was extracted from six ovaries of each group with TRIzol reagent according to the manufacturer’s instructions (Life Technologies, Carlsbad, CA, USA). Total RNA concentration was evaluated by gel electrophoresis and spectrophotometer (METTLER, TOLEDO, Canada), respectively. Total RNA was used as a template for reverse transcription to produce cDNAs with the Prime Script TM RT reagent Kit with gDNA Eraser (Stratagene, Takara, San Jose, CA, USA). 

qRT-PCR was conducted on the MSLPCR30 Thermal Cycler system (Biobase Biozone Co., Ltd., Guangdong, China) and β-actin was used to normalize the expression values. The specific primers used in this study are listed in Table 1.

### 2.7. Protein Extraction and Western Blot Analysis

Total protein was obtained from the homogenates of 6 ovaries from each group by lysing in 800 μL of lysis buffer (7 M urea, 2 M thiourea, 2% DDT, 4% CHAPS, 20 mM Tris base, 1% protease inhibitor cocktail, 0.5 µL benzonase, and 20 µL/mL Bio-Lytes3/10, Fudebio-Tech, China). After centrifugation at 12,000× *g* round per minute (RPM) for 20 min at 4 °C, the supernatant was collected carefully. The procedure was managed according to previous reports [37]. After electrophoresis and electrotransfer, Nylon Fluoride (PVDF) membranes (Millipore, Burlington, MA, USA) were cut, blocked against Bovine Serum Albumin (5% BSA, AUG pharma, Giza, Egypt), resolved in Tris Buffered Saline with Tween (TBST) before one hour and then blotted with primary antibodies all night.

Procedures for all antibodies were performed following the manufacturer’s instructions; recombinant anti-mutant P53 antibody (1:1000 dilution, Abcam, ab32049, Cambridge, UK), anti-VASA/VAS antibody (1:500 dilution, Abcam, ab209710, Cambridge, UK), anti-progesterone receptor Pgr (1:1000 dilution, Abcam, ab75857, Cambridge, UK), recombinant anti-Caspase-3 p12 antibody Caspase-3 (EPR16888) (1:1000 dilution, Abcam, ab179517, Cambridge, UK), and anti-β-actin antibody -N- terminal (1:500 dilution, Abcam, ab209869, Cambridge, UK), separately. Membranes were then incubated with horseradish peroxidase (HRP)-linked secondary antibody (1:5000) and visualized using enhanced Western blotting detection reagents (Amersham Pharmacia Biotech, London, UK). 

Image J software (Version number (1.51K), National Institutes of Health, Rockville, MD, USA) was used for protein quantifications and blot scanning. These procedures were conducted in the Faculty of Medicine, Mansoura University, Mansoura, Egypt.

### 2.8. Statistical Analysis and Graph Preparation 

Data were analyzed using GraphPad Prism8 software (8.00, GraphPad Software, Inc., La Jolla, CA, USA) and reported as the mean ± standard error of the mean (SEM). Differences in ovarian anomaly and ZnO-NPs residue accumulation were analyzed using a one-way analysis of variance (ANOVA), followed by Tukey’s multiple comparisons test. A value of * *p* < 0.05 was considered significant.

## 3. Results

### 3.1. Zinc Oxide Nanoparticles (ZnO-NPs) Characterization

**Figure 1:**
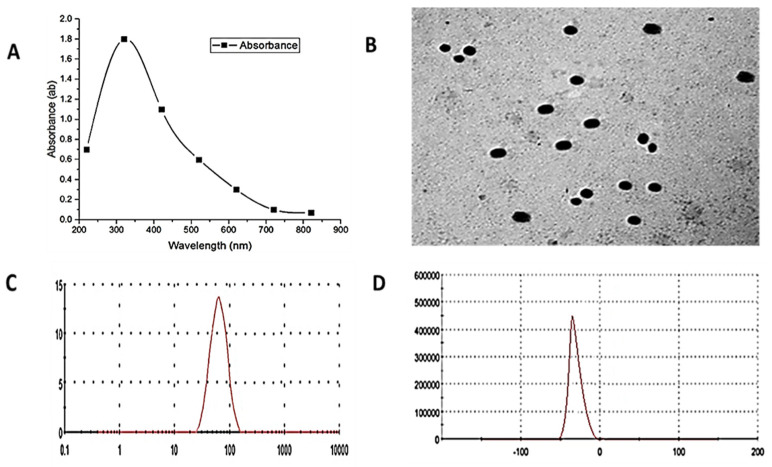
ZnO-NPs characterization: (**A**) shows the UV–VIS spectroscopy analysis of ZnO-NPs to estimate their optical properties; the maximum peak was 340 nm. (**B**) The size and shape of the ZnO-NPs were spherical with an average size of 108 nm by TEM. (**C**) DLS analysis showed that the exact size was 89 nm. (**D**) Zeta potential analysis showed that the net surface charge of ZnO-NPs was −33 mV.

The UV-visible absorption spectra of ZnO-NPs are shown in Figure 1A. The samples of ZnO-NPs have a strong absorption maximum at 340 nm.

Moreover, TEM measurements showed good distribution of ZnO-NPs, which had a spherical shape, an average size of 108 nm, and presented no agglomeration (Figure 1B). 

DLS analysis of particle size distribution revealed an average particle size of around 89 nm (based on intensity distribution) (Figure 1C). The ZnO-NPs had a mean zeta potential of −33 mV, indicating moderate stability (Figure 1D). 

### 3.2. ZnO-NPs Affect Ovarian Morphology and Alter Female Fertility and Fecundity 

**Figure 2:**
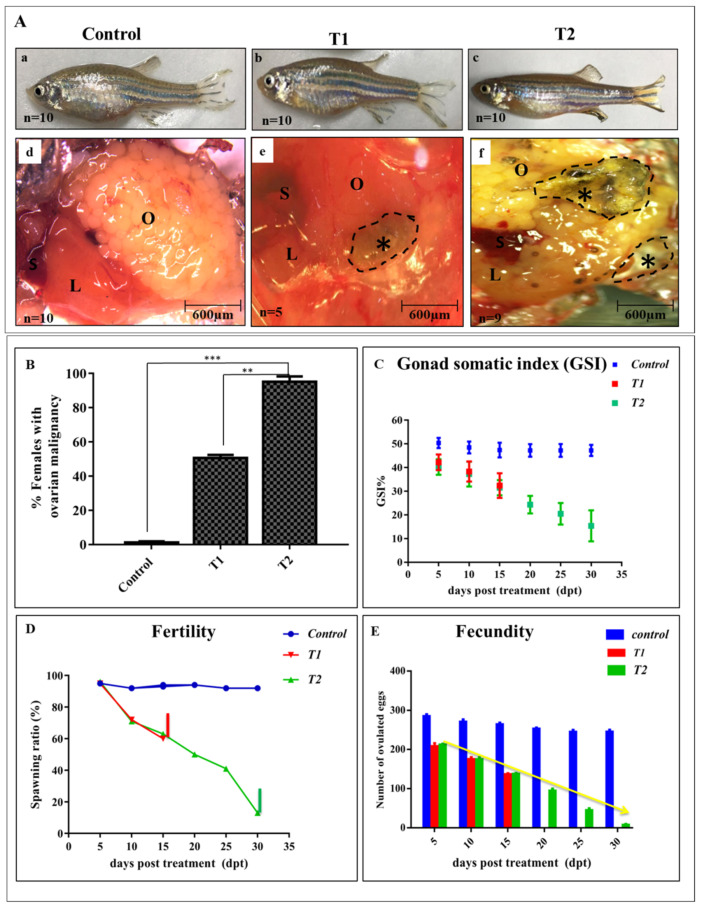
Effects of ZnO-NPs on Gonadosomatic index, fertility, and fecundity: (**A**) Representative pictures of the control, T1, and T2 females with normal ovaries in the control fish (**a**,**d**), transition state of malignancy in T1 (**b**,**e**), and ovarian necrosis in T2 (**c**,**f**) (Black asterisks). (**B**) Percentage of females with macroscopic ovarian malignancy for each experimental group (*n* = 10). (**C**) Distribution of gonad somatic index (GSI); notice the weight reduction of T2 females compared with the control. (**D**) Fertility assessment of the control, T1, and T2 groups declined at 30 dpt in T2 into 10%. (**E**) Tracing assessment of fecundity in the control, T1, and T2 with a significant reduction in T2 at 30 dpt. L: liver, O: ovary, S: spleen. The results are shown as the mean ± SEM. ** *p* < 0.01, *** *p* < 0.001. T1 = group was exposed to 1/5th of estimated LC_50_ of ZnO-NPs in water daily for two weeks (15 days). T2 = group was exposed to 1/5th of estimated LC_50_ of ZnO-NPs in water daily for one month (30 days).

Females of the control group grew normally and did not manifest any ovarian abnormalities (Figure 2A(a,d)), whereas 50% of T1 females showed mild differences from the control upon dissection (Figure 2A(b,e)). On the other hand, 90% of the dissected treated females from T2 had diffused empty ovaries with confocal macro-necrosis compared with the matched control and T1 (Figure 2A(c,f)). At the experimental end, the percentage of females with ovarian malignancy was recorded (Figure 2B).

During the exposure period, it was noticed that ZnO-NPs affected the females’ body weight; accordingly, the GSI was reduced significantly after one-month post-ZnO-NPs exposure rather than two weeks (Figure 2C). 

Moreover, fertility and fecundity assessments were conducted every five days and the spawned eggs were calculated after mating with the males. After one month of ZnO-NPs exposure, the females’ fertility declined and nearly all of the treated females were infertile by 30 dpt compared with the control females (Figure 2D). Furthermore, the number of spawned eggs declined sharply after one month in the T2 females compared with the control (Figure 2E). 

### 3.3. Zn Residues in the Female’s Body

The tissue analysis of Zn residues in the whole body of the fish is represented in Table 2. 

Herein, the highest concentration of Zn was detected in the body tissue of females that were exposed to ZnO-NPs for one month, followed by fish exposed to ZnO-NPs for two weeks, then the control group that showed the lowest value of Zn residues. 

### 3.4. Effect of ZnO-NPs on Histological Findings of Oocyte Maturation Levels 

**Figure 3:**
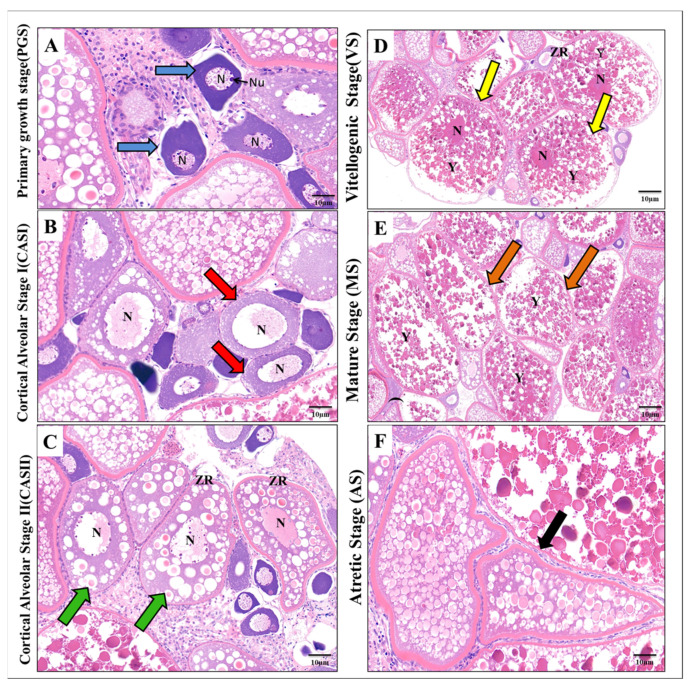
Histological structure showing the different stages of oocyte development in the control zebrafish ovary: (**A**) Primary growth stage (PGS) (blue arrow). (**B**) Cortical Alveolar Stage I (CASI) (red arrow). (**C**) Cortical Alveolar Stage II (CASII) (green arrow). (**D**) Vitellogenic Stage (VS) (yellow arrow). (**E**) Mature Stage (MS) (orange arrow). (**F**) Atretic Stage (AS) (black arrow). N: nucleus, Nu: nucleolus, Y: yolk, ZR: Zona radiata (H&E, 10 µm).

After one month, the ovaries of the control females had normal histological architecture and contained different stages of oocytes development, including primary oocytes or the primary growth stage (PGS), cortical alveolar stage I (CAS I), predominantly vitellogenic stage (VS), mature stage (MS), and atretic oocytes. In the primary oocytes stage, the oocyte was small, and spherical, with a central large nucleus containing deeply stained nucleoli at the peripheral zone, and the ooplasm was more intense compared with other stages (Figure 3A). The cortical alveolar stage I (CAS I) was characterized by the presence of cortical alveoli cells that gradually grew and began to fill the ooplasm around the central nucleus to differentiate into the cortical alveolar stage II (CASII) (Figure 3B,C). 

The nucleoli at the CASII stage-oriented periphery migrated to the center of the nucleus, and at the same time, the zona radiata became well-developed (Figure 3C). As a consequence, the number of yolk granules increased in the ooplasm to give rise to the vitellogenic stage (VS) (Figure 3D). 

At this stage, the nucleus was difficult to observe and the oocytes enlarged gradually and entered into the mature stage (MS), in which the oocyte was filled with large vitellus yolk granules. In this stage, the nucleus completely disappeared due to the intense diffusion of yolk granules (Figure 3E). Normally, ovarian follicles can either grow to mature oocytes or breakdown by a process of follicular atresia (a form of normal apoptosis to control the oocyte number in the ovarian pool); at this stage, the atretic oocytes (AS) were determined by the hypertrophy of granulosa and theca cells with irregularity in the zona radiata (Figure 3F). 

**Figure 4:**
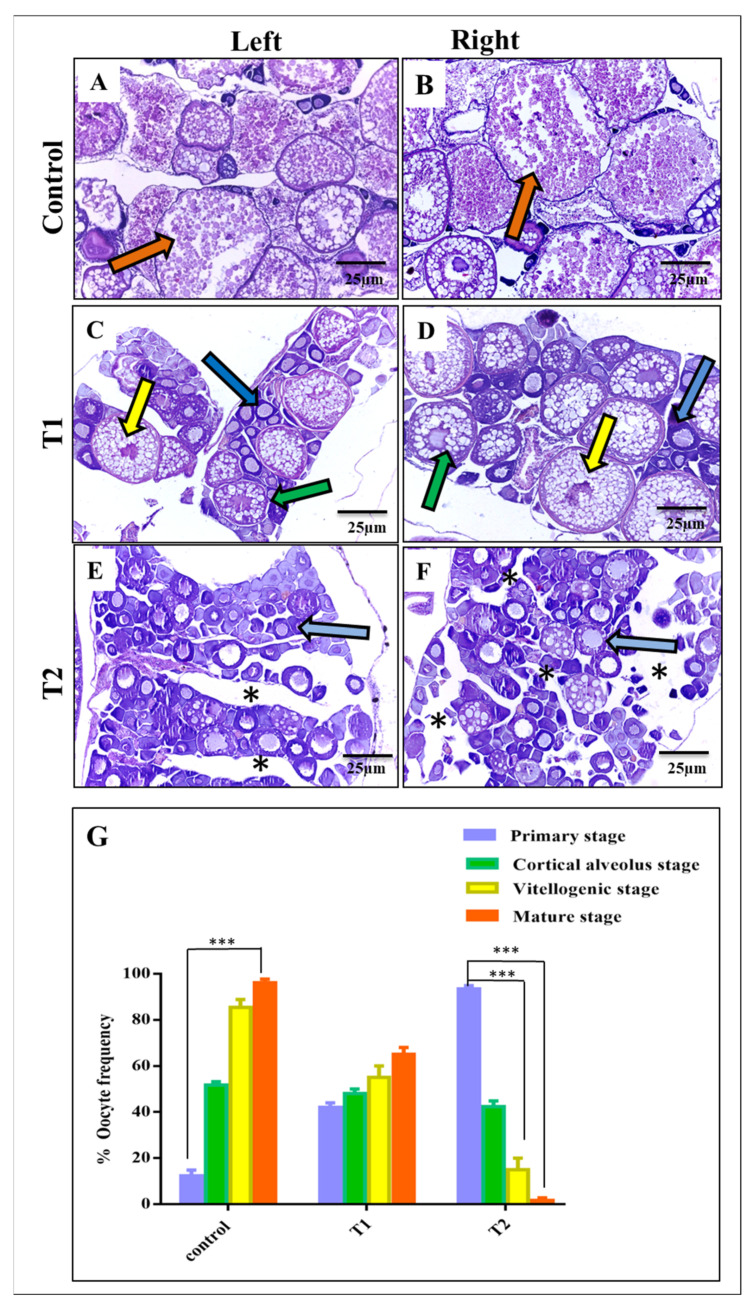
Comparative histological observations of the control, T1, and T2 left and right ovaries (H&E, 25 µm)**:** (**A**,**B**) Photomicrographs showing the left and right ovaries of the control; notice the presence of numerous mature oocytes (orange arrow). (**C**,**D**) Photomicrographs showing the left and right ovaries of T1; notice the presence of the primary growth stage (blue arrow), cortical alveolar stage II (green arrow), vitellogenic stage (yellow arrow), and the absence of the mature stage. (**E**,**F**) Photomicrographs showing the left and right ovaries of T2; notice the tissue degeneration (black asterisk) and the prominent distribution of primary oocytes (blue arrows). (**G**) Statistical analysis of ovary sections in the three experimental groups. Data are shown as the mean ± SEM. *** *p* < 0.001. T1 = group was exposed to 1/5th of estimated LC_50_ of ZnO-NPs in water daily for two weeks (15 days). T2 = group was exposed to 1/5th of estimated LC_50_ of ZnO-NPs in water daily for one month (30 days).

Compared with the control ovaries (Figure 4A,B), the T1 females revealed a significant reduction in the number of mature oocytes, however cortical and vitellogenic stages were still present (Figure 4C,D). Contrarily, females of the T2 group exhibited abnormal ovarian structure with a significant tissue degeneration and a dominant distribution of primary oocytes. Here, cortical, vitellogenic, and mature stages were rarely observed and nearly absent in some sections (Figure 4E,F). Statistical analysis of the histological sections confirmed the previous observations and revealed the adverse consequences of ZnO-NPs on the developing granulosa cells around the nucleus of the oocytes that affected oocyte maturation and differentiation (Figure 4G).

### 3.5. ZnO-NPs Induce Cytotoxicity and Autophagosome Formation at the Ultrastructural Level 

**Figure 5:**
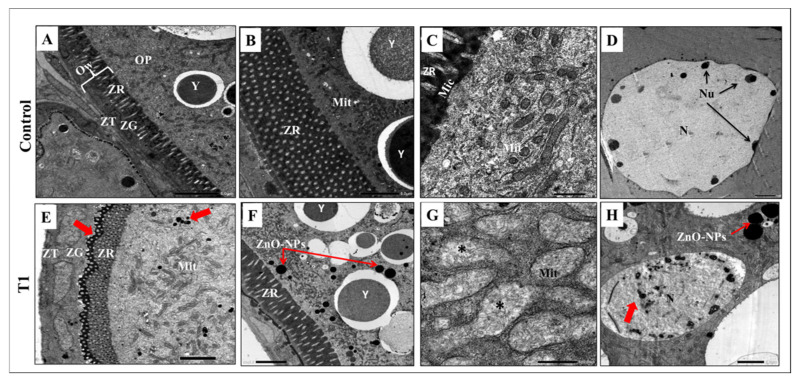
Transmission Electron Microscopy observation of the control and T1 ovaries: (**A**–**D**) Transmission electron micrographs of the control vitellogenic oocyte showing the intact oocyte membrane with distinct cell layers of theca cells (ZT), granulosa cells (ZG), and zona radiata (ZR), large yolk granules, plenty of normal mitochondria, and a normal nucleus. (**E**–**H**) Transmission electron micrographs of the T1 ovary showing diffusion of ZnO-NPs to the ooplasm through the cell membrane (red arrows); notice the mild alteration of the cell membrane, mitochondrial degeneration, and chromatin condensation. OW: oocyte wall, ZT: Zona theca, ZG: Zona granulosa, ZR: Zona radiata, OP: ooplasm, Y: yolk, Mit: Mitochondria, Mic: microvilli, N: nucleus, Nu: nucleolus. T1 = group was exposed to 1/5th of the estimated LC50 of ZnO-NPs in water daily for two weeks (15 days).

The control-group oocyte’s TEM analysis is displayed in Figure 5A–D. There were three main layers in the contact wall; zona theca (ZT) contained theca cells, zona granulosa (ZG), which included the granulosa cells, and zona radiata (ZR) that appeared as a complex perforated layer crossed by pores or canals contain oocyte microvilli. Zona radiata (ZR) was the main feature of the previtellogenic oocyte (Figure 5A). The ooplasm of the previtellogenic oocyte was rich with large yolk granules and mitochondria that had quite regular cisternae (Figure 5B,C). The nucleus had an intact regular nuclear envelope and many small nucleoli arranged in the peripheral zone at the nuclear membrane (Figure 5D).

Contrarily, oocytes revealed mild alterations to the cell wall, ooplasm, and nucleus after two weeks of ZnO-NPs exposure in the T1 group. Herein, the zona radiata (ZR) manifested an irregular appearance with diffused ZnO-NPs that appeared as black droplets penetrating the zona radiata to the ooplasm (Figure 5E,F). In the ooplasm, there were signs of ruptured mitochondrial cisternae (Figure 5G). In addition, the nucleus had a state of chromatin condensation and diffused nuclear material (Figure 5H). 

**Figure 6:**
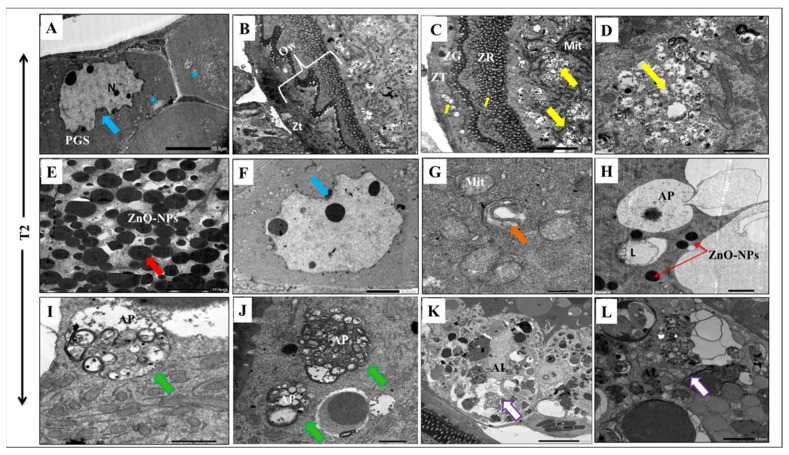
Transmission Electron Microscopy observation of T2 ovary: (**A**) Transmission electron micrographs of a primary oocyte (PGS) showing abnormal winding nuclear membrane (blue arrow) and cytoplasmic vocalizations (blue asterisks). (**B**–**G**) Micrographs of vitellogenic oocytes in abnormal conditions exhibited irregular cell walls with some vacuolization in the granulosa cell layer and the ooplasm (yellow arrow) with complete organelle degeneration (yellow arrow) due to the enormous diffusion of ZnO-NPs (red arrow). The nucleus appeared with a winding nuclear membrane (blue arrow) and the mitochondria exhibited atrophic cisternae and a swollen appearance (orange arrow). (**H**–**L**) Transmission electron micrographs showing the steps of autophagy; notice the presence of numerous autophagosomes (green arrow) and autolysosomes (white arrow). OW: oocyte wall, ZT: Zona theca, ZG: Zona granulosa, ZR: Zona radiata, N: nucleus, Mit: Mitochondria, PGS: primary growth stage, AP: autophagosome, AL: autolysosome.

On the other hand, the long exposure to ZnO-NPs for 30 days had significant cytotoxic effects on the developing oocytes. The primary oocyte had a large, indented nucleus with an irregular envelope and separate nuclear components (Figure 6A). The observed vitellogenic oocyte manifested apoptotic features with a winding oocyte wall (Figure 6B). Moreover, the long ZnO-NPs exposure led to ooplasm vacuolization with organelle degeneration due to the high diffusion of the nanoparticles (Figure 6C,E). The nucleus appeared with an irregular tortuous membrane (Figure 6F), and the mitochondria had fragmented cisternae and a swollen appearance (Figure 6G). Interestingly, long exposure to ZnO-NPs activated macroautophagy (also termed autophagy) and altered the intracellular degradation system in the cytoplasm (Figure 6H,I). Here, the ooplasm of T2 contained numerous autophagosomes that were double-membrane vesicles that contained degenerated cellular organelles (Figure 6J). The process of macroautophagy was completed when the autophagosomes fused with the lysosomes to form autolysosomes where the inner cellular cargo degraded (Figure 6K,L).

### 3.6. Effect of ZnO-NPs on the Expression of Specific Genes (Antioxidant, Autophagy, Apoptosis, Oocyte Maturation, and Ovulation-Related Genes) in Zebrafish Ovarian Cells 

**Figure 7:**
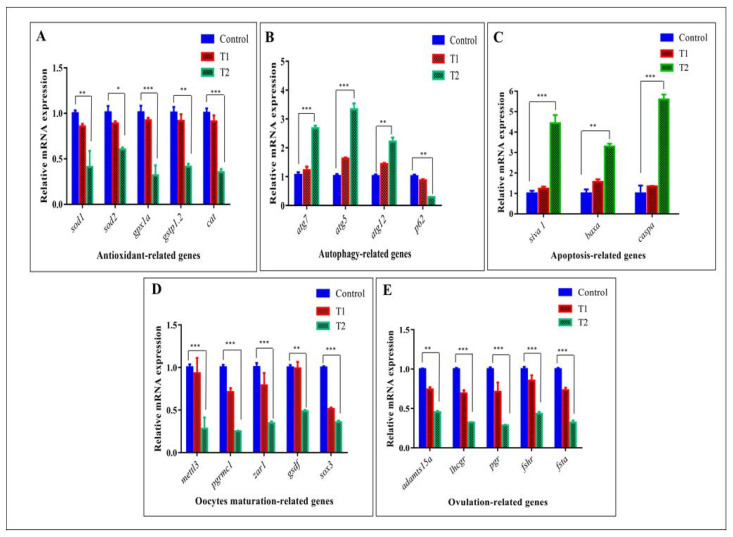
Effect of ZnO-NPs on oocyte maturation and ovulation via the induction of oxidative stress and the apoptotic pathway at the genetic level. (**A**–**C**) Expressions of mRNA evaluated by qRT-PCR indicate the downregulation of antioxidant-related genes in T2 (**A**), the upregulation of autophagy-related genes in T2 (**B**), and the induction of apoptosis-related genes in T2 (**C**). (**D**,**E**) mRNA expression shows the downregulation of oocyte maturation-related genes in T2 (**D**) and ovulation genes in T2 (**E**). The results from three dependent experiments are shown as the mean ± SEM. * *p* < 0.05, ** *p* < 0.01, *** *p* < 0.001. T1 = group was exposed to 1/5th of the estimated LC50 of ZnO-NPs in water daily for two weeks (15 days). T2 = group was exposed to 1/5th of the estimated LC_50_ of ZnO-NPs in water daily for one month (30 days).

TEM observations were supported by epigenetic expression evaluated by qRT-PCR. Herein, in T2, signals from the damaged mitochondria increased the ROS from the endoplasmic reticulum which led to high oxidative stress (OS). ROS activated DNA damage and autophagy pathways capable of inducing cell death (apoptosis). Accordingly, mRNA expression of antioxidant genes (*sod1*, *sod2*, *gpx1a*, *gstp1.2*, and *cat*) declined in the T2 group compared with the control and T1 groups (Figure 7A).

On the contrary, mRNA expression of autophagy genes (*atg7*, *atg5*, and *atg12*) was gradually increased in the T2 group and involved in the autophagosome formation, whereas *p62*, the autophagic cargo receptor, gradually declined, thus indicating the actual autophagy activation (Figure 7B). 

In addition, mRNA expression of apoptosis-related genes was elevated in the T2 group compared with the control and T1 siblings via a higher expression of *siva*, *bax*, and *caspa* (Figure 7C). 

Most likely, oxidative stress, autophagy, and apoptosis induction were responsible for the oocyte ovulation and maturation suppression in the T2 ovary indicated by the downregulation of mRNA expression of oocyte maturation-related genes including *mettl3*, *pgrmc1*, *zar1*, *gsdf*, and *sox3*, that in turn affected the ovulation process via the suppression of ovulation-related genes (*adamts15a*, *ihcgr*, *pgr*, *fshr*, and *fsta*) (Figure 7D,E). 

### 3.7. Effect of ZnO-NPs on the Expression of Proteins Related to Apoptosis Genes 

**Figure 8:**
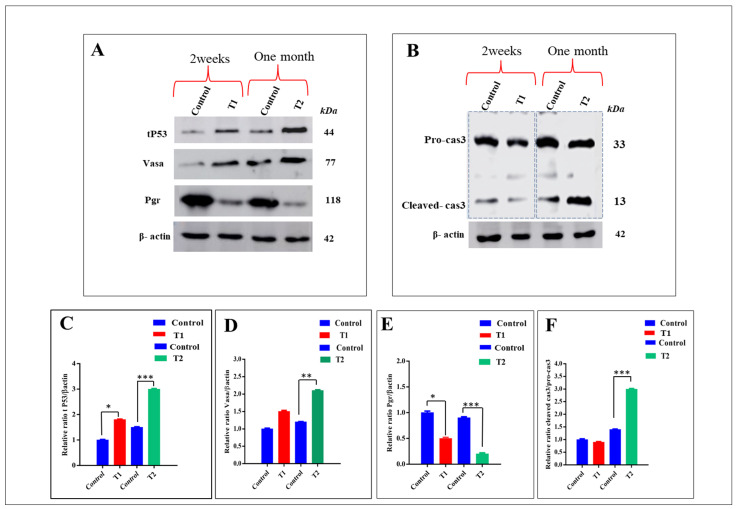
Effect of ZnO-NPs on necroptosis via mutated tP53 formation: (**A**) Western blot analysis of the tP53 protein, Vasa protein, and Pgr protein normalized to β-actin in control, T1, and T2 groups. (**B**) Western blot analysis of procaspase-3 and cleaved caspase-3 in the control, T1, and T2 groups revealed the overexpression of cleaved caspase-3 in T2 compared with the control and T1 groups. (**C**–**F**) Quantified protein expression revealed the ratio analysis of tP53/β-actin, Vasa/β-actin, Pgr/β-actin, and cleaved -cas3/pro-cas3, respectively quantified by the Image J software. Data expressed as mean ± SEM. * *p* < 0.05, ** *p* < 0.01, and *** *p* < 0.001. T1 = group was exposed to 1/5th of the estimated LC_50_ of ZnO-NPs in water daily for two weeks (15 days). T2 = group was exposed to 1/5th of the estimated LC_50_ of ZnO-NPs in water daily for one month (30 days).

To confirm the relationship between ZnO-NPs aggregation and necrotic tissue formation in the T2 ovary, a Western blot was performed on the ovaries of the control, T1, and T2 groups that revealed a higher accumulation of mutated **tP53** protein in the T2 group relative to the control and T1 siblings (Figure 8A,C). Mutated tP53 protein marked the damaged DNA and favored cancer cell survival. Furthermore, the T2 group exhibited a higher expression of the **Vasa** protein that was specific for germ cells and primordial gametocytes, indicating that ZnO-NPs did not affect germ cell differentiation, ensuring the presence of many primary oocytes in the T2 ovaries rather than the control and T1 groups (Figure 8A,D). On the other hand, the T2 group exhibited a significant reduction of the progesterone receptor protein **Pgr**, which plays an important role in the ovulation process (Figure 8A,E). 

In addition, ZnO-NPs induced the apoptosis of oocytes via the caspase pathway, revealed by the higher expressions of the **Cleaved-cas3** protein in the T2 group compared with the control and T1 (Figure 8B,F).

## 4. Discussion

In relation to the characterization of ZnO-NPs, their strong absorption at 340 nm was firstly confirmed by Dulta *et al*., who reported that the maximum absorption of ZnO-NPs was in the range of 300–360 nm, with a very high peak at 340 nm [38]. The absorption spectra of produced nanoparticles are influenced by a variety of factors, including synthesis technique, temperature, size, and shape [39]. This strong absorption refers to the spherical shape of the NPs as well as the monodispersed nature of their distribution, which is confirmed by the results of the TEM measurements [40,41]. Because of the electrostatic repulsions between the individual particles, a large negative or positive value of zeta potential suggests a higher physical colloidal stability [42].

During the exposure period to ZnO-NPs, the ZnO-NPs affected the females’ body weight; in fact, the resulting GSI significantly reduced after one month rather than after two weeks. Kime attributed the decrease in GSI to vitellogenesis suppression [43]. Vitellogenesis is the production and secretion of vitellogenins (Vtgs) from the liver as primary precursors of yolk proteins in response to the estrogen stimulation [44]. ZnO-NPs have been shown in studies to produce intracellular reactive oxygen species (ROS) [45,46], which damage biomolecules, such as DNA, lipids, and proteins in the liver and ovaries, as well as other organs, and hence hinder the vitellogenesis process [43,45,47]. 

Collectively, over time, ZnO-NPs alter female fertility and fecundity. The ZnO-NPs hampered the growth of the zebrafish embryo and larva and reduced their survivability and hatching rate, where the 84 h half-maximum effective concentration (EC_50_) of ZnO-NPs on the hatching rate of zebrafish embryo was 2.065 mg/L [48]. ZnO-NPs reduced the hatching rate and caused pericardial edema of zebrafish embryos and larvae possibly by increasing ROS and altering the cellular response to oxidative stress [9]. ZnO-NPs delayed zebrafish embryo and larva development, decreased their survival and hatching rates, and caused serious tissue damage and ulcerations [10].

The current results agree with those of Tavabe et al., who found that ZnO-NPs have a dose-dependent effect on *Macrobrachium rosenbergii* egg weight, viable egg rate, and broodstock inter-spawning period [49]. Wang, et al. also noted that chronic exposure (13 weeks) to zebrafish of 0.1 mg L-1 titanium dioxide nanoparticles (nTiO_2_) led to a reduction of 29.5% in the total egg number [50]. The NPs can overcome biological barriers and directly alter primary ovarian follicles, as well as subsequently affect the zebrafish vitellogenesis [50]. Tang et al. demonstrated that ZnO-NPs’ detrimental effect on reproductive organs was generated by apoptosis and signaling in the endoplasmic reticulum [51], while Bacchetta, et al. attributed the reduced reproduction in *Daphnia magna* to a direct injury to the mitochondria, resulting in poor energy availability [52].

The detection of Zn residues in the female’s body agrees with a previous work that found the accumulation of Zn resulting from waterborne exposure to ZnO-NPs to be 7 to 12 times higher in blackfish than in the control group [53]. Kaya, et al. found that 14 days of waterborne exposure by *Oreochromis niloticus* to small (30 nm) and large (100 nm) ZnO-NPs led to a significant accumulation of ZnO-NPs in the tissues [54]. On the contrary, the tissues of zebrafish exposed to ZnO-NPs at 500 μg/L or 5000 μg/L via the water revealed no significant uptake of zinc in the studied tissues evaluated (liver, gill, kidney, and brain) [55]. The physicochemical properties of NPs, as well as the exposure method and fish species, alter tissue distribution patterns and accumulation, which could explain the disparity in results [56].

For instance, Suganthi, et al. observed ovary histopathological abnormalities in *Oreochromis mossambicus* after exposure to ZnO-NPs for 4 days [57]. The histopathological abnormalities of *O. mossambicus* ovaries differed depending on the concentration of ZnO-NPs, where degenerated late vitellogenic oocytes (DLV) and the development of vacuolation (V) in the cytoplasm were seen in 30 ppm-exposed fish. Oocyte necrosis and vitellogenic fluid (Vf) in the ovarian parenchyma were seen at 50 ppm. Degenerated late vitellogenic oocytes (DLV) and vacuolation (V) production in the cytoplasm were detected at 70 ppm. In addition, it has been observed that the ZnO–NPs (50 mg/L) could induce more substantial histological damages to adult zebrafish ovaries than perfluorooctane sulfonate (PFOS) alone [58]. According to Liu et al., ZnO NPs have a detrimental effect on ovarian development because of their effects on neuronal factors, neuroendocrine cells, or the contents of essential elements found in the ovary [59]. On the other hand, mitotic catastrophe, vacuolization in the ooplasm, enlargement of the mitochondria, and degradation of the pore and microvilli structures of the zona radiata were recorded in zebrafish that were exposed to titanium dioxide nanoparticles (TiO_2_-NPs) for 5 days [60]. Numerous studies support the histological impact of TiO_2_-NPs on ovarian anomalies in zebrafish [50,61]. 

The most significant of several hypotheses suggests that TiO_2_-NPs have a degenerative effect on folliculogenesis, alter sex hormone levels and decrease the fertility [50,61]. Moreover, the TEM study of mouse OGCs subjected to ZnO-NPs revealed a reduction of cell viability in a concentration- and time-dependent manner. 

Treatment with 10 g/mL ZnO-NPs for one day was non-toxic, while the structures of the cells treated with 30 g/m ZnO-NPs for a day were disrupted [11]. Interestingly, a study performed in testicular cells revealed that ZnO-NPs increased the numbers of autophagosomes and caused an aberrant enlargement of the nuclear membrane intermembrane gap [62].

Moreover, a quantitative transcriptomics (RNA-seq) study on gene expressions in ovarian samples showed that a total of 222 genes were altered following the ZnO-NP treatments [59].

Numerous studies link ZnO-NPs’ cytotoxic effects to oxidative stress development and the increase of ROS levels in the cells, which disrupts the cellular redox balance and can seriously harm biological macromolecules and cause genetic instability [63,64,65]. 

In mouse OGCs subjected to 20 and 30 g/mL ZnO-NPs, it was detected that ROS production caused a decrease in the viability of the OGCs [12]. Similarly, Chen et al. recorded that gene expressions associated with oxidative stress (Gclc) levels in the placentas of pregnant mice were downregulated after exposure to ZnO-NPs [66]. 

This downregulation resulted in impairing the ability of the body to effectively eliminate ROS, particularly H_2_O_2_, which led to an increase in oxidants and oxidative stress causing fetus damage and fetal abortions.

According to several studies, ZnO-NPs can trigger the autophagy process in cells by releasing zinc ions that harm lysosomes and disrupt autophagic flux and mitochondria [67,68]. The autophagy signaling system is controlled by a crucial molecular signal called mTOR. 

More than 20 genes, including the Atg5, Atg7, and atg12 genes, can encode the proteins necessary for the autophagy field execution [69]. Numerous works have shown that ZnO-NPs can increase the expression of atg genes, particularly atg5 and atg7, and the proteins involved in the production of the autophagosome [68,70,71].

Similarly, in cultured primary astrocytes, it was discovered that ZnO-NPs activated caspase-3 and caused the production of the bax gene [72]. 

This was supported by Ahamed et al., who demonstrated that ZnO-NPs triggered apoptotic cell death by inducing p53 and caspase pathways through the action of the oxidative stress [73]. 

Additionally, Chen et al. reported that the silver nanoparticles (Ag-NPs) can trigger the maturation of zebrafish oocytes by inducing apoptosis in ovarian follicle cells as a result of oxidative stress [74]. While, according to Applerot et al., the lysosomal cells initially absorbed the nano-ZnO and then released Zn^2+^ [75]. 

This is consistent with Mishra et al. who stated that ZnO-NPs triggered apoptosis and the production of ROS [76].

Our results suggested that long exposure to ZnO-NPs maintains primary oocyte dormancy and the few growing oocytes died before complete maturation, hence ceasing the ovulation process. Within the normal female ovary, hormonal action has a key role in controlling oocyte maturation and ovulation, however, it depends on the cross-talk between other cellular and molecular mechanisms including autophagy, apoptosis, and necroptosis that also determine the fate of the oocytes [77,78]. 

Accordingly, as long as the autophagy process is stable, the other two pathways are controlled to maintain fertility for zebrafish females [23,79]. In addition, progesterone receptor membrane component 1 (Pgrmc1) plays an essential role in promoting the survival of both the normal and cancerous ovarian [80,81]. Pgrmc1 expression has been correlated with tumor growth, angiogenesis, and infertility [82,83]. Oocyte maturation is essential for the ovulation step and any disturbance will affect the fertility [84]. Furthermore, incomplete maturation alters ovulation and follicular-stimulating genes [85]. 

As for the effects of ZnO-NPs on the expressions of proteins related to apoptosis-related genes, the Western blot revealed a higher accumulation of mutated tP53 protein in the T2 group relative to the control and T1 siblings. Mutated tP53 protein marked the damaged DNA favoring cancer cell survival. Furthermore, the T2 group exhibited a higher expression of the Vasa protein that is specific for germ cells and primordial gametocytes, indicating that ZnO-NPs did not affect germ cell differentiation, ensuring the presence of many primary oocytes in T2 ovaries rather than in the control and T1 groups. 

On the other hand, the T2 group exhibited a significant reduction of progesterone receptor protein Pgr, which has an essential function in the ovulation process. In addition, ZnO-NPs induced oocyte apoptosis via the caspase pathway, which was revealed by the higher expression of cleaved -cas3 protein in the T2 group compared with the control and T1.

Moreover, the ovaries of the T2 females harboring high levels of the tP53-mutated protein that has been reported to be increased in half of the solid tumors, induced cancer cells to survive [86,87]. 

In zebrafish, the Vasa protein is involved in germ cell formation and is stable throughout early development [88], however, its relation to ovarian necrosis is still poorly understood. Here, we found that the expression of the Vasa protein is upregulated in T2 females compared with the control, supporting the previous results observed in other ovarian cancers [89,90,91]. 

## 5. Conclusions

In conclusion, our results revealed that ZnO-NPs can impair the fertility of female zebrafish in a time-dependent manner. Exposing zebrafish to 1/5th of the estimated LC_50_ of ZnO-NPs (0.69 mg/L) daily for one month caused cytotoxicity in the maturing oocyte by activating autophagy and apoptosis in a caspase-dependent manner and induced oxidative stress by generating ROS that elevated the mutated ovarian tP53 protein. These effects could be implicated in delaying the development of embryos and inducing embryonic abnormalities. 

## Figures and Tables

**Table 1:** Target genes, accession number, and primer sequences for quantitative real-time PCR (qRT-PCR).

Target Gene	Accession Number in NCBI	Primer Sequences
**Reference Gene**		
βactin	NM_131031	F: 5′ ATGGATGAGGAAATCGCTGC 3′
		R:5′CTTTCTGTCCCATGCCAACC 3′
**Antioxidant enzymes**		
Superoxide dismutase1 (*sod1*)	NM_131294	F: 5′ CGCACTTCAACCCTCATGAC 3′
		R: 5′ TGAATCACCATGGTCCTCCC 3′
Superoxide dismutase2 (*sod2*)	NM_199976	F:5′CCTCCAGACAGAAGCA 3′
		R:5′CTGAAATGAGCCAAAGT 3′
Glutathione peroxidase 1a (*gpx1a*)	NM_001007281	F:5′GCACAACAGTCAGGGAT 3′
		R:5′TCAGGAACGCAAACAG 3′
Glutathione S-transferase pi 1.2 (*gstp1.2*)	NM_131734	F:5′CCAACCACCTCAAATGCT 3′
		R:5′ACGGGAAAGAGTCCAGACAG 3′
Catalase (*cat*)	NM_130912	F:5′TGTGGAAGGAGGGTCG 3′
		R:5′CTTTGGCTTTGGAGTAG 3′
**Autophagy-related genes**		
Autophagy-related gene-7 homolog (*atg7*)	XM 021479676	F:5′ACGGTGATGCTGTTGGTCTG 3′
		R: 5′ TTTGTCGGTGGATTTGAAGG 3′
Autophagy-related gene-5 homolog (*atg5*)	NM_205618	F: 5′ TGGAGTATCCCACCGAAGA3′
		R:5′ CACTGGTCGGAAGAGC3′
Autophagy-related gene-12 homolog (*atg12*)	NM_001246200	F: 5′ TCATCTCACGCTTCCTCAA 3′
		R: 5′ TCACTTCCGAAACACTCAAA 3′
Sequestosome 1 (sqstm1) (*p62*)	NM_001312913	F: 5′ TGGTGCTACTGCCTCTTCTCA 3′
		R: 5′ GGGTTACTTTGGTCCGCTTT 3′
**Apoptosis-related genes**		
Apoptosis-inducing factor (*siva1*)	NM_001327928	F: 5′ CCGCTACCGACAGGAGATCTACGA 3′
		R: 5′ GGTGTGGAGCGCGCTCTGTGCAGT 3′
BCL2 associated X, apoptosis regulator (*baxa*)	NM_131562	F: 5′ GACAGGGATGCTGAAGTGA 3′
		R: 5′ TGAGTCGGCTGAAGATTAGA 3′
Caspase a (*caspa*)	NM_131505	F: 5′ GACGGTGAGCCTGATGAGCCAA 3′
		R: 5′ CCTGAACAGTTCCTCGATGTGA 3′
**Oocyte maturation genes**		
Methyltransferase like 3 (*mettl3*)	NM_212780	F: 5′ CCTAGAGCTGCTGAATACCAGT 3′
		R: 5′ GATGATTCGCCTGAAGTGC 3′
Progesterone receptor membrane component-1 (*pgrmc1*)	NM_001007392	F: 5′ CAGACTATGGCCCGGTTGAGGAG 3′
		R: 5′ CTGCATGGCATTGAGATCGG 3′
Zygote arrest 1 (*zar1*)	NM_194381	F: 5′ CAACCCGAAGACCGAC 3′
		R: 5′ CACCACCGCTGCTGAC 3′
Gonadal soma-derived factor variant 2 (*gsdf*)	NM_001114668	F: 5′ GCTCCATCCGTCACCT 3′
		R: 5′ TCACCGTAGACAGAACCAG 3′
SRY-box transcription factor 3 (*sox3*)	NM_001001811	F: 5′ ATTCCGCAGTCCAACA 3′
		R: 5′ TTCTCCTGAGCCATCTTC 3′
**Ovulation-related genes**		
Metallopeptidase with thrombospondin type 1 motif, 15a (*adamts15a*)	NM_001126429	F:5′GAGAGCAAAGATAACAAGGCACAAA3′
		R: 5′TTTTCCACCTTTATTGACTCCACCT3′
Luteinizing hormone/choriogonadotropin receptor (*lhcgr*)	NM_205625	F: 5′ CGCTCTGATCAACTGGGACA 3′
		R: 5′ GGCGCTGTTGGCATAAATCC 3′
Progesterone receptor (*pgr*)	NM_001166335	F: 5′ ACAGACAGCATACACCGC3′
		R: 5′TCCACAGGTCAGAACTCC3′
Follicle-stimulating hormone receptor (fshr)	NM_001001812	F: 5′ CAAATGCGTCTACGCCATGC3′
		R: 5′AAAGCGGGATTACGGACGGT3′
Follistatin a (*fsta*)	NM_131037	F: 5′ CATCAAGGCCAAGTCATGCG3′
		R: 5′GCCTGCTTCATGGCACACTC3′

**Table 2:** Accumulation of ZnO-NPs residues in the whole body of treated female zebrafish (µg/g wet weight).

Groups	Zn Residues (μg/g Wet Weight)
control	15.83 ± 1.57 ^c^
T1	32.10 ± 4.01 ^b^
T2	57.85 ± 1.78 ^a^
SEM	6.26
*p*-value	<0.001

Means within columns carrying different superscripts are significant at (*p* < 0.05). T1 = group was exposed to 1/5th of estimated LC_50_ of ZnO-NPs in water daily for two weeks (15 days). T2 = group was exposed to 1/5th of estimated LC_50_ of ZnO-NPs in water daily for one month (30 days).

## Data Availability

The data are contained within this article.
